# Comparative Metabolomic Analysis Reveals the Role of *OsHPL1* in the Cold-Induced Metabolic Changes in Rice

**DOI:** 10.3390/plants12102032

**Published:** 2023-05-19

**Authors:** Ziwei Wu, Zhiyu Guo, Kemiao Wang, Rui Wang, Chuanying Fang

**Affiliations:** 1 Sanya Nanfan Research Institute, Hainan University, Sanya 572025, China; 2 School of Tropical Crops, Hainan University, Haikou 570288, China

**Keywords:** lipoxygenism, hypothermia, JA, metabolic reprogramming

## Abstract

Cytochrome P450 (*CYP74*) family members participate in the generation of oxylipins and play essential roles in plant adaptation. However, the metabolic reprogramming mediated by *CYP74s* under cold stress remains largely unexplored. Herein, we report how cold-triggered *OsHPL1*, a member of the *CYP74* family, modulates rice metabolism. Cold stress significantly induced the expression of *OsHPL1* and the accumulation of OPDA (12-oxo-phytodienoic acid) and jasmonates in the wild-type (WT) plants. The absence of *OsHPL1* attenuates OPDA accumulation to a low temperature. Then, we performed a widely targeted metabolomics study covering 597 structurally annotated compounds. In the WT and *hpl1* plants, cold stress remodeled the metabolism of lipids and amino acids. Although the WT and *hpl1* mutants shared over one hundred cold-affected differentially accumulated metabolites (DAMs), some displayed distinct cold-responding patterns. Furthermore, we identified 114 and 56 cold-responding DAMs, specifically in the WT and *hpl1* mutants. In conclusion, our work characterized cold-triggered metabolic rewiring and the metabolic role of *OsHPL1* in rice.

## 1. Introduction

Cold stress is a major environmental stress impairing plant growth and limiting the productivity of crop plants [1,2]. Thus, plants have evolved various adaptive strategies toward cold stress, including metabolic reprogramming [3,4]. Previous studies showed that cold stress increased many metabolites, including amino acids (asparagine, aspartate, glycine, and proline), organic acids (ascorbate, gluconate, malate, and α-ketoglutarate), and carbohydrates (sucrose, maltose, glucose, fructose, and trehalose) [5,6]. Notably, some of these compounds have been found to play a role in enhancing cold tolerance. For example, lipids and amino acids have been widely reported to be involved in cold-stress responses [7,8]. The cell membrane is the leading site of cold-induced injury. When there is cold stress, plants remodel lipid metabolism and protect membrane fluidity [9]. During the cold-stress response, plant cells accumulate low-molecular-weight osmoregulation metabolites, such as proline. These compounds protect plants from alleviating osmotic stress and maintaining cell swelling, water absorption, and metabolic activities [1,2]. A comparative metabolomic analysis covering 223 metabolites demonstrated that the prominent metabolic responses were centered on antioxidation during cold treatment [3]. In addition, most flavanols and anthocyanins accumulate during cold exposure, and the decrease in flavonoid content impairs the freezing tolerance of leaves. Flavonoid metabolism plays a vital role in the freezing tolerance of Arabidopsis [10]. 

Cytochrome P450 (CYP74) family members participate in various biochemical processes, such as the generation of oxylipins. Oxylipin synthesis starts mainly from the polyunsaturated C18 fatty acids: oxygenations transfer linolenic (18:3) and linoleic (18:2) acids to hydroperoxides. Then, CYP74s work to produce allene oxides, divinyl ethers, or short-chain aldehydes. The corresponding enzymes are allene oxide synthase (AOS), divinyl ether synthase [11], or hydroperoxide lyase (HPL). The AOS branch fluxes into the (+)-cis-12-oxo-phytodienoic acid (OPDA) and jasmonate [12] biosynthesis pathway, generating jasmonic acid (JA) and derivates [13]. HPLs catalyze hydroperoxides into aldehydes and oxoacids, major green leaf volatiles. According to substrate specificities, HPLs are classed into 13-HPL (CYP74B) and 9-/13-HPL (CYP74C). The 13-HPLs cleave only 13-hydroperoxides, while the 9-/13-HPLs have activity toward both 9- and 13-hydroperoxides. These reactions are essential in plant defense and signaling [14,15,16]. The rice genome contains two *OsAOS* genes and three *OsHPL* genes. Both *OsAOSs* contribute to the biosynthesis of JA [17,18]. *OsHPL1* and *OsHPL2* belong to the *CYP74C* subfamily and metabolize 9- and 13-hydroperoxides of linoleic and linolenic acid into aldehydes [19], while OsHPL3 is a 13-HPL protein [16]. Despite the lack of in vivo evidence, biochemical data show that *OsHPL1* and *OsHPL2* also have AOS activity [19].

Oxylipins are essential for plant adaptivity to environmental stresses. Jasmonates, the best-characterized oxylipins, can modulate a range of physiological, biochemical, and molecular processes to help plants cope with cold temperatures [20,21]. In Arabidopsis, cold stress induces the expression of JA biosynthetic genes, which leads to the accumulation of JA, thereby enhancing cold tolerance [22]. The exogenous application of jasmonate significantly improved the cold tolerance of plants to cold acclimation [23]. Recent studies have shown that impaired biosynthesis or signaling of jasmonic acid (JA) can significantly impair the freezing tolerance of Arabidopsis. Jasmonate-ZIM domain (JAZ) proteins, which respond to jasmonic acid, repress AtICE1 (INDUCER OF CBF EXPRESSION 1)-mediated cold tolerance. Cold stress triggers the synthesis of jasmonates and the degradation of JAZ proteins. As a result, the released ICE1 activates downstream genes to protect Arabidopsis plants against cold stress [23,24]. The cold-induced accumulation of jasmonates is conserved in Arabidopsis and rice [23,25]. A QTL study for cold tolerance in rice deciphered that HAN1 confers cold tolerance in rice. HAN1 reduces cold tolerance by converting JA-Ile to the inactive form 12-hydroxy-JA-Ile (12OH-JA-Ile). Functional nucleotide polymorphism in the promoter leads to enhanced transcription of HAN1 in japonica, contributing to its adaptation to a temperate climate during northward expansion [26]. In addition, environmental temperature changes also affect the emission of the HPL branch-derived volatiles in tomato [27]. However, the function of HPLs in cold responses in rice remains obscure.

Expression patterns of *CYP74s* suggested *OsHPL1′*s responses to cold stress. We also found *OsHPL1* was crucial for OPDA accumulation under cold stress. Then, we performed widely targeted metabolomics and characterized the metabolic rewiring in WT rice under cold treatment. Then, we compared metabolic responses to cold in the *hpl1* mutants and WT plants. Our work outlined the role of *OsHPL1* in regulating cold-induced metabolic reprogramming in rice.

## 2. Results

### 2.1. Expression Analysis of CYP74s under Cold Stress

**Figure 1:**
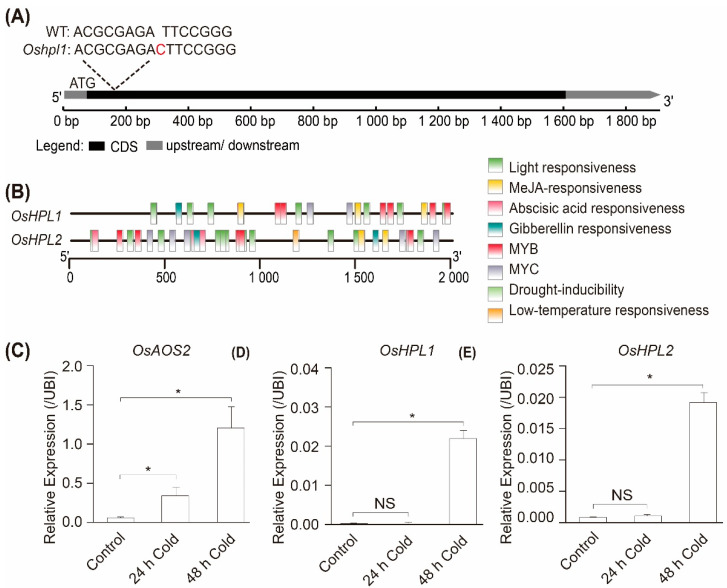
Analysis of *OsHPL1* genes in rice. (**A**) Base sequence of *OsHPL1*. (**B**) The response elements in the promoter of *OsHPL1* and *OsHPL2*. (**C**–**E**) The relative expression was analyzed by qRT–PCR with *UBIQUITIN (UBI)* endogenous control. The data were presented as mean ± SD of three biological replicates; * indicates *p* < 0.05, NS indicates no significant difference, Student’s *t*-test.

We analyzed expression patterns to examine the cold responsiveness of cytochrome P450 family members in rice. Cold stress significantly triggered the expression of *OsAOS2 (LOC_Os03g12500)*, *OsHPL 1(LOC_Os02g12690)*, and *OsHPL2 (LOC_Os02g12680)*, while *OsAOS1* (LOC_Os03g55800) was undetectable (Figure 1C–E). We also investigated the cis-elements present in the promoters of the *CYP74s. OsHPL1′*s promoter contains more than 150 response elements, including low-temperature and MeJA response elements (Figure 1B). These findings suggest that *OsHPL1* may participate in the cold response of rice.

### 2.2. Impaired HPL1 Attenuates JAs’ Responses to Cold Stress

**Figure 2:**
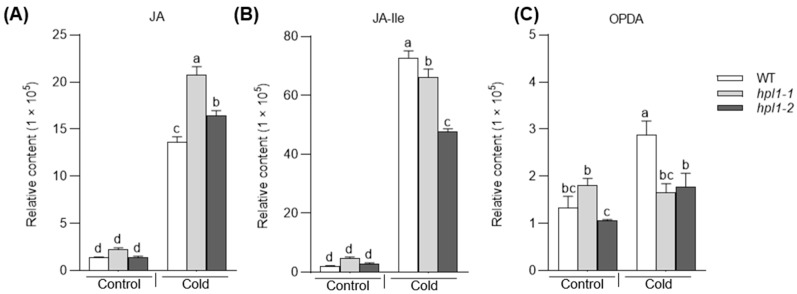
The abundance of JA (**A**), JA-lle (**B**), and OPDA (**C**) in *hpl1* mutants and wild-type plants. The data were represented as mean ± SD of three biological replicates; in each plot, bars with the same lowercase letter are not significantly different (*p* < 0.05). WT and *hpl1* mutants were stored at normal and cold conditions for 24 h (Control and Cold). JA represents jasmonic acid, OPDA represents 12-oxo-phytodienoic acid, and JA-Ile represents Jasmonic Acid-Isolacine.

To characterize the response of the jasmonate pathway to cold stress, we analyzed the contents of JAs in wild-type (WT) rice exposed to a low-temperature treatment (6 °C). A 24 h treatment significantly induced the accumulation of OPDA, JA, and JA-Ile in the WT plants (Figure 2A–C and Table S3). 

Considering the pivotal role of *CYP74s* in the biosynthesis of oxylipins, we wondered whether the absence of *OsHPL1* in rice affects JA accumulation and subsequent response to cold. We obtained two loss-of-function mutants, *hpl1-1* and *hpl1-2*, which carried a 1-bp insertion downstream of the start codon (Figure 1A). Without cold stress, the WT and the mutant plants showed comparable levels of OPDA, JA, and JA-Ile. Resembling that in WT plants, JA and JA-Ile increased in *hpl1* mutants under cold stress. However, *hpl1* mutants showed no significant changes in OPDA content under cold stress (Figure 2A–C, Tables S3 and S4). To summarize, cold stress triggers the jasmonate pathway in rice, while impaired *OsHPL1* attenuates OPDA’s responses to low temperature.

### 2.3. Cold Triggers Metabolic Rewiring in WT Rice Plants

We conducted widely targeted high-throughput LC-MS/MS analyses under normal and cold conditions to draw a whole picture of cold-triggered metabolic reprogramming. In total, we detected a total of 713 metabolites, including 597 structurally annotated compounds. These included both primary and secondary metabolites: (i) lipids accounted for the most significant proportion, followed by amino acids and their derivatives, and nucleotides and their derivatives; (ii) secondary metabolites mainly include 122 flavonoids, 33 phenolamines, and 23 terpenoids (Table S2).

**Figure 3:**
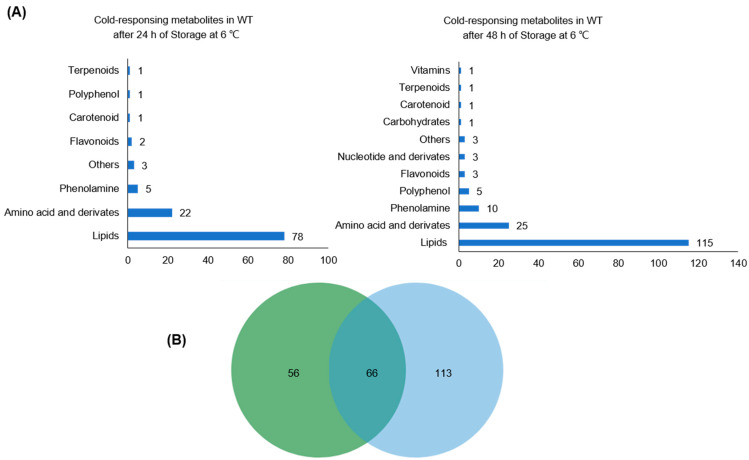
A comparative analysis of metabolites involved in cold response in wild-type plants. (**A**) Distribution of metabolite species of WT. The pie chart shows cold-responding metabolites in WT after 24 h of Storage at 6 °C (**left**) and 48 h at 6 °C (**right**). These metabolites were divided into 8 (**left**) and 11 (**right**) categories, mainly including lipids, flavones, and amino acids and derivatives. (**B**) Venn diagram shows the number of DAMs in WT under different cold treatment times.

Next, we performed a comparative analysis to identify cold-responding metabolites in WT plants. Compounds with a 2-fold change (*p* < 0.05) in the contents between the control and cold stress were annotated as differentially accumulated metabolites (DAMs). As a result of a 24 h cold stress, 113 DAMs belonging to eight categories were affected, comprising 68 up-regulated and 45 down-regulated compounds (Figure 3A and Table S3). Upon a 48 h treatment, 131 cold-induced and 37 cold-repressed DAMs spanning 11 categories were characterized (Figure 3A and Table S3). Notably, over 80% of the DAMs were lipids (64% and 68% under 24 h and 48 h cold stress, respectively) and amino acids and their derivatives (16%, 14%) (Figure 3B). 

Detailed analysis of the DAMs revealed different expression patterns between 24 h and 48 h cold treatment. Specifically, we observed that 38 up-regulated and 10 down-regulated metabolites were shared between the two treatments. However, while a 24 h cold treatment led to the repression of 13 compounds, including 12 lipids and N-cinnamoyl-tryptamine, a 48 h cold treatment induced their production.

**Figure 4:**
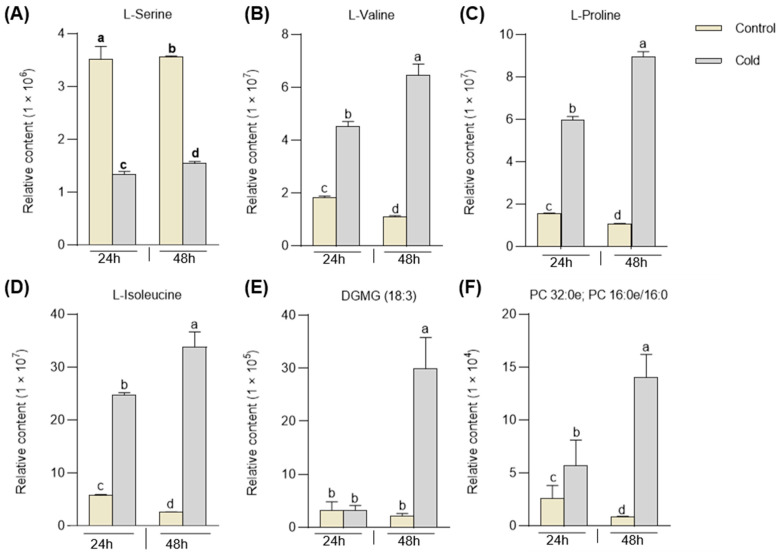
The abundance of metabolites WT. Histogram shows the abundance of amino acids (**A**–**D**) and lipids (**E**,**F**) in WT that were stored at normal and cold conditions for 24 h and 48 h (Control and Cold). The data were represented as mean ± SD of three biological replicates; in each plot, bars with the same lowercase letter are not significantly different (*p* < 0.05). DGMG represents digalactosylmonoacylglycerol; PC represents phosphatidylcholine.

Moreover, most differentially accumulated lipids and phenolamines (69% and 60%, respectively) were cold-induced. Interestingly, only L-serine and cystathionine among the 29 cold-responding amino acids and derivatives declined under stress (Figure 4A,B). At the same time, the levels of L-proline, L-valine, and L-isoleucine increased both after a 24 h and 48 h cold treatment, with a more significant increase observed in the latter (Figure 4C–E and Table S3). Notably, the response to cold stress of specific compounds. For instance, although the levels of DGMG (18:3) and PC 32:0e; PC 16:0e/16:0 showed minimal increases following 24 h of cold treatment, their content exhibited a significant rise after 48 h of cold treatment (Figure 4F and Table S3).

### 2.4. The Effects of OsHPL1 on Rice Metabolomes 

**Figure 5:**
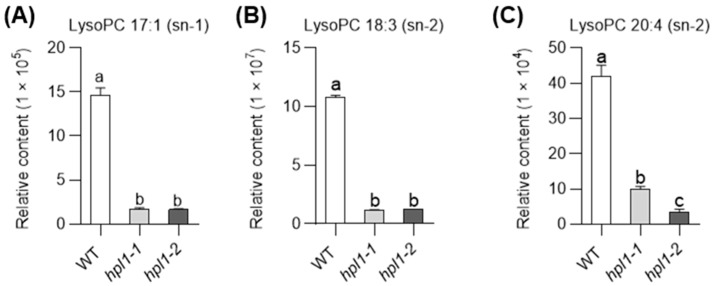
The abundance of lysoPC 17:1 (sn-1) (**A**), lysoPC 18:3 (sn-2) (**B**), and lysoPC 20:4 (sn-2) (**C**) in *hpl1* mutants and WT under normal conditions for 24 h (Control). The data were represented as mean ± SD of three biological replicates; in each plot, bars with the same lowercase letter are not significantly different (*p* < 0.05). LysoPC represents lyso-phosphatidylcholine.

To investigate the metabolic role of *OsHPL1*, we performed a comparative analysis in *hpl1-1* and *hpl1-2*. Compared with the WT plants, the mutants accumulated higher levels of lipids, specifically fatty acids (FAs) and lysophospholipids. Meanwhile, the loss of *OsHPL1* repressed the production of 65 metabolites, including lipids, amino acids and derivatives, and flavonoids (Tables S4 and S5). Remarkably, lipid-related metabolites constituted a significant proportion of the declined metabolites, accounting for nearly 87% of the total. Lysophospholipids were noted to be the most prominent among them, including lysoPC 17:1 (sn-1), lysoPC 18:3 (sn-2), and lysoPC 20:4 (sn-2) (Figure 5A–C, Tables S4 and S5). These data suggest a role of *OsHPL1* in the rice metabolome, especially in the lipid pathway.

**Figure 6:**
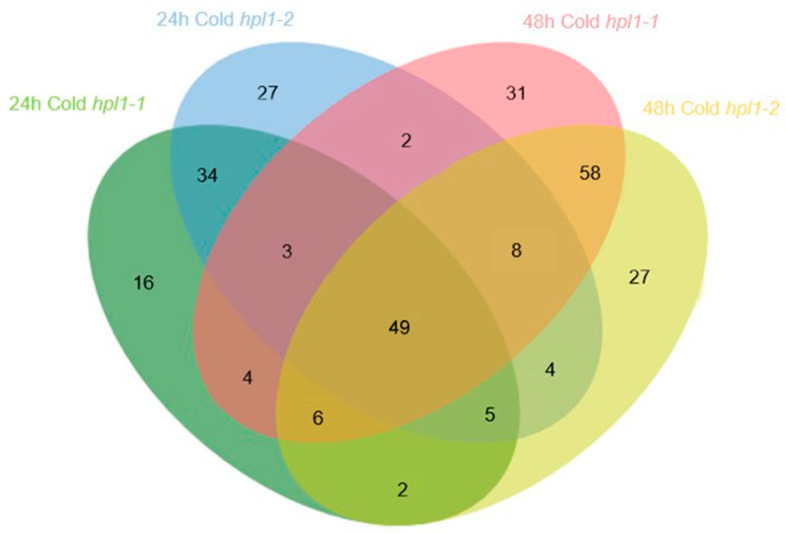
Schematic representation of metabolites with altered accumulation levels of *hpl1* mutants. Venn diagram of DAMs in *hpl1* mutant under control and cold condition.

Then, we analyzed the cold-triggered metabolic rewiring in the mutants. The two *hpl1* mutants shared 49 cold-responding DAMs after being treated for 24 h and 48 h. Of these, lipids accounted for 37, whereas amino acids and derivatives accounted for the remaining 10 (Figure 6, Tables S4 and S5). In total, 143 compounds piled up after the cold treatment, including >98% lipids and >92% amino acids (Tables S4 and S5). 

**Figure 7:**
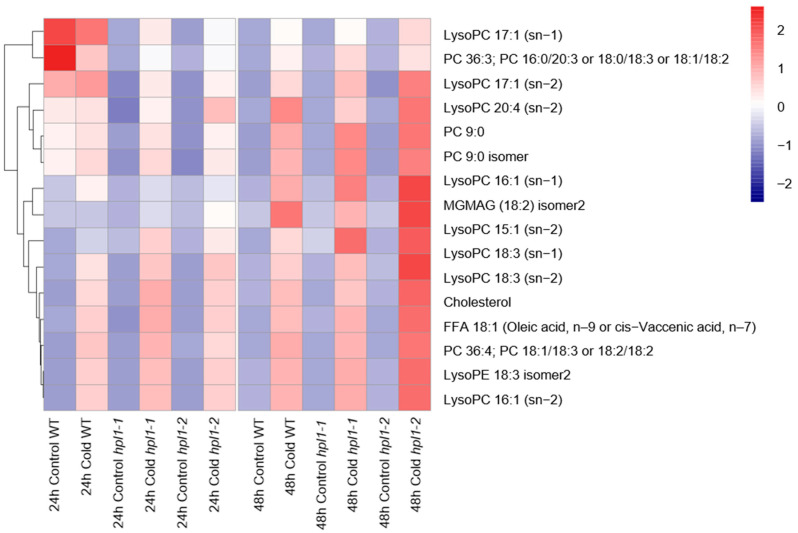
Accumulation of lipids in *hpl1* mutants and WT. Heat map shows the abundance of 16 lipids in *hpl1* mutants and WT that were stored at normal and cold conditions for 24 h and 48 h (Control and Cold). FFA represents free fatty acids; LysoPC represents lyso-phosphatidylcholine; PC represents phosphatidylcholine; LysoPE represents lyso-phosphatidylethanolamine.

To further define *HPL1′s* role in cold-triggered metabolic responses, we analyzed the differences in cold-responding DAMs between the *hpl1* mutants and WT plants. The WT and *hpl1* mutants shared 106 cold-responding DAMs after being treated for 24 h/48 h. Among them, 79 and 21 compounds were lipids, and amino acids and their derivatives, respectively. Detailed analysis revealed distinct patterns of the commonly identified DAMs in different genotypes. Specifically, sixteen lipids, including nine lysoPCs/lysoPEs, displayed cold-repressed patterns in the WT, whereas their content increased significantly in the *hpl1* mutants after cold stress (Figure 7, Tables S3–S5). 

We identified 114 and 56 cold-responding DAMs specifically in the WT and *hpl1* mutants, respectively (Tables S3–S5). Conserved in the WT and the mutant plants, lipids accounted for the most significant proportion of genotype-dependent cold-regulated DAMs. 

**Figure 8:**
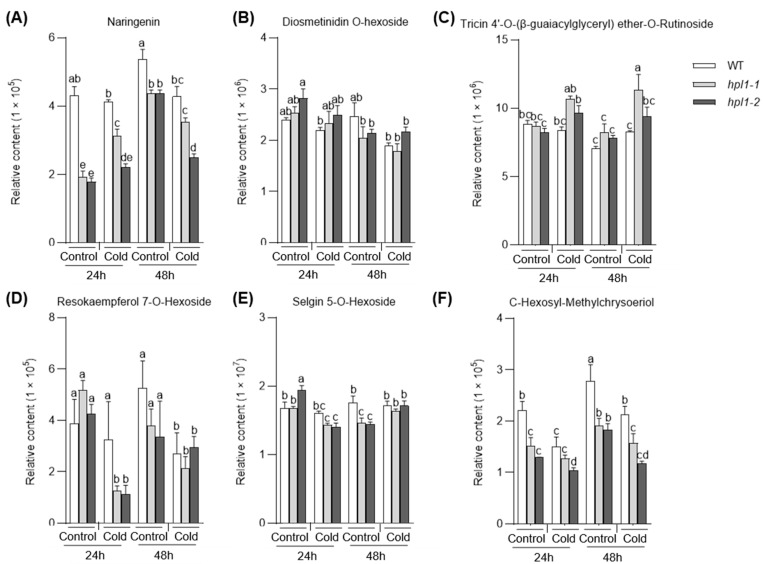
The abundance of cold induced (**A**–**C**) and depressed (**D**–**F**) flavonoids in *hpl1* mutants and WT under normal and cold conditions. The bar plots show the abundance of cold-responsive flavonoids in WT and *hpl1* mutants that were stored at normal and cold conditions for 24 h and 48 h (Control and Cold). The data were represented as mean ± SD of three biological replicates; in each plot, bars with the same lowercase letter are not significantly different (*p* < 0.05).

Moreover, flavonoids also responded to cold stress differently in the WT and *hpl1* mutants. In the *hpl1* mutants, five and three flavonoids, none overlapping with those in the WT, were induced (Figure 8A–C) and depressed (Figure 8D–F) by cold, respectively.

## 3. Discussion

Oxylipins are essential in plants’ responses and adaptations to environmental stresses, including cold stress. As a *CYP74* family member, *OsHPL1* has 9-/13-HPL and AOS activity [17]. However, the in vivo role of *OsHPL1* in metabolism and cold responses remains unknown. In this study, we obtained mutants of *OsHPL1* and performed a comparative analysis with metabolome data. Our work revealed *OsHPL1′s* role in cold-triggered metabolic rewiring.

The oxylipins pathway starts with converting linolenic (18:3) and linoleic (18:2) acids to hydroperoxides. Then, phylogenetically related yet distinct *CYP74* members divide the metabolic flux into different branches. AOSs catalyze the production of jasmonates, while HPLs lead to the generation of green leaf violates. *OsHPL1* and *OsHPL2* expressed in E. coli cleaved 9- and 13-hydroperoxide of linoleic and linolenic into aldehydes, releasing C6 and C9 violates. In addition, *OsHPL1* and *OsHPL2* also have limited AOS activity [28]. *OsHPL1* and *OsHPL2* share 84% of their amino acid residues and show comparable biochemical activity [28]. In rice plants, the white-backed planthopper infestation activates *OsHPL2* expression and (E)-2-hexenal production. Overexpression of *OsHPL2* enhances the emission of (E)-2-hexenal and (2E,6Z)-nonadienal, which are produced by 13-HPL and 9-HPL, respectively [29]. The in vivo data of rice plants conform the 9-/13-Hydroperoxide Lyase function of *OsHPL2*. This study found that *OsHPL1* regulates lipid metabolism and jasmonate production, suggesting potential crosstalk between HPL and AOS branches. The observation in cea62, a mutant *OsHPL3* with 13-HPL activity, further supports this possibility. A premature stop codon in *OsHPL3* depressed the release of C6 violates and triggers the overproduction of JA. JA biosynthetic and signaling genes are expressed at significantly higher levels in the cea62 mutant than in the WT plants [16]. Since AOSs compete for substrates with HPLs, the activation of JA synthesis in cea62 may result from the remodeling metabolic flux. Alternatively, the HPL branch may regulate the AOS branch through a signal transduction pathway.

Our work characterized the metabolic reprogramming of rice under cold stress. Cold treatment has a significant impact on the accumulation of numerous metabolites belonging to different classes, with more than 60% of these being cold-induced (Figure 3). For instance, most amino acids and derivatives built up upon cold stress, which is consistent with a previous report [3]. Moreover, lipid remodeling is important in cold tolerance, confirmed in the model plant A. thaliana, algae, and several crops. There are specific changes among various plants [30,31]. Our data also showed clear evidence of lipid metabolism reprogramming under cold stress. The content of most lysophospholipids increased after cold treatment. In addition, *hpl1* mutants accumulated more lysophospholipids than WT plants, suggesting the role of *OsHPL1* in cold-regulated lipids. It has been reported that the biosynthetic pathways of vitamin E and vitamin K1 form a subnetwork, which is responsible for japonica and indica cold tolerance divergence [32]. Although we also detected alpha-tocotrienol, its contents were comparable in the WT and mutants.

Knowledge about the modulation of cold stress on the phenylpropane pathway is limited. Recent work has reported phenolamine responses to cold in Poa crymophila [33]. Our work revealed that cold stress regulates the phenylpropane pathway, such as flavonoids. Despite the effects of cold on the flavonoid pathway, changed compounds in the WT and *hpl1* mutants were distinct. These suggest potential roles of *HPL1* in the cold-regulated phenylpropane pathway. However, the detailed function of the mechanisms of flavonoids’ responses to cold remains to be elucidated.

Moreover, *HPLs* are expressed with distinct patterns. Although *OsHPL1* is ubiquitously expressed, the expression of *OsHPL2* is limited to the leaves and leaf sheaths. Meanwhile, *OsHPL3* shows leaf-specific and wound-inducible expression patterns [28]. The distinct expression patterns indicate different roles of HPL*s* in plant development and adaptation. Our findings demonstrate that *OsHPL1* and *OsHPL2* exhibit similar responses to cold stress. Thus, whether the two *CYP74C* members work redundantly in cold responses remains to be elucidated.

## 4. Materials and Methods

### 4.1. Plant Materials

The CRISPR/Cas9-mediated gene editing mutants for *hpl1 (LOC_Os02g12690)* were obtained from Biogle Genome Editing Ctr [34]. The rice plants and their background material, japonica rice variety ZH11, were cultivated at Hainan University (Haikou, China, 20°02′ N, 110°11′ E). All the seeds were germinated for three days at 37 °C on filter paper soaked in distilled water and then planted in seedbeds. Subsequently, two-week-old seedlings were planted by hydroponic culture using Yoshida nutrient solution [35]. 

### 4.2. RNA Extraction and Expression Analyses

In this study, one-month-old seedlings were utilized to collect RNA samples under normal growth conditions and after exposure to cold stress (6 °C for 24 and 48 h). Leaves from three separate seedlings were harvested and rapidly frozen in liquid nitrogen. Approximately 100 mg of powdered samples were subjected to RNA extraction using a previously described protocol [35]. Total RNA was extracted using an RNA extraction kit (TRIzol reagent; Invitrogen, Carlsbad, CA, USA) following the manufacturer’s instructions. Specifically, 3 μg of RNA was used to synthesize first-strand cDNA in a 20 μL reaction mixture with the EasyScript One-Step gDNA Removal and cDNA Synthesis SuperMix (TransGen, Beijing, China). Quantification of transcript abundance was conducted using the SYBR Premix Ex Taq kit (TaKaRa, Tokyo, Japan) on the ABI 7500 Real-Time PCR system (Applied Biosystems, Foster City, CA, USA), with expression levels normalized to the expression of the rice *UBIQUITIN* (LOC_Os03g13170). Specifically, the relative expression level of the target gene was determined using the 2^−ΔCt^ method, where ΔCt represents the difference in Ct values between the target gene and the reference gene *UBIQUITIN*. RT–qPCR analyses were performed for three biological replicates, and primer information is provided in Table S1.

### 4.3. Bioinformatic Prediction of the OsHPL1 Promoter Using PlantCare

The *OsHPL1* promoter sequence was analyzed using the PlantCare software (http://bioinformatics.psb.ugent.be/webtools/plantcare/html/, accessed on 22 March 2022).

### 4.4. Metabolic Sample Preparation 

In metabolic analyses, one-month seedlings were used, and the leaves were sampled and extracted under normal conditions and after cold treatment (6 °C for 24 h and 48 h, respectively). Leaves were harvested from *hpl1* mutants and WT plants into 1 mL centrifuge tubes and quickly frozen in liquid nitrogen [36]. Samples from three independent plants were combined to form one biological replicate for metabolite extraction. Three biological replicates were collected from each genotype.

### 4.5. Metabolomic Detection

The freeze-dried samples were ground using a grinder (MM 400, Retsch, Haan, Germany) operated at 30 Hz for 1.5 min, and the resulting powder was collected in a 2 mL centrifuge tube. Subsequently, approximately 100 mg of the powdered samples were weighed and mixed with 70% methanol aqueous solution at 0.1 mg/mL. The mixture was extracted by ultrasonication at 40 Hz for 10 min. After centrifugation and filtration (SCAA-104, 0.22 mm pore size; ANPEL, Shanghai, China), the supernatant was quantified by the MRM method of LC-MS 8060 (Shimadzu, Kyoto, Japan) [37,38,39], with the detection window set to 120 s and the target scan time to 1.5 s. A total of 713 transitions were monitored, and the original data were processed by Multiquant 3.0.2.

### 4.6. The Analysis of Differentially Accumulated Metabolites (DAMs)

The metabolites’ contents were normalized by dividing the relative signal strengths of the metabolites by the strength of the internal standard (0.1 mg/L lidocaine). Then, log_2_ transformed the values to improve the normalization further. The identification criteria of differential metabolites were |log_2_ (fold change)| > 1 and *p*-value < 0.05, which was calculated by univariate analysis (*t*-test) [36]. Nested ANOVA calculated differences between the metabolites in the hpl1 mutants and WT in Excel 2010 and GraphPad Prism 8. The Venn plots illustrating the shared DAMs in the *hpl1* mutants and WT were generated using the online tool available at http://jvenn.toulouse.inra.fr/app/index.html (accessed on 12 March 2023).

## 5. Conclusions

In this study, we comprehensively analyzed the metabolic flexibility of cold stress in rice. Our work characterized the effects of *OsHPL1* in oxylipin-included lipid metabolism and its responses to cold. In addition, we also identified the participation of *OsHPL1* in the cold-triggered rewiring of the phenylpropane pathway, especially flavonoids. While the detailed molecular mechanisms require further exploration, our findings offer novel insights into *OsHPL1′s* function in metabolic adaptation under cold stress.

## Data Availability

Data is contained within the article or supplementary material.

## Supplementary Materials

The following supporting information can be downloaded at: https://www.mdpi.com/article/10.3390/plants12102032/s1. Table S1: The primers used in this study; Table S2: List of the metabolites detected in this study; Table S3: The content of cold-related DAMs in WT at 24 and 48 h of cold treatment; Table S4: The content of cold-related DAMs in *hpl1-1* at 24 and 48 h of cold treatment; Table S5: The content of cold-related DAMs in *hpl1-2* at 24 and 48 h of cold treatment.
